# STAT proteins: a kaleidoscope of canonical and non-canonical functions in immunity and cancer

**DOI:** 10.1186/s13045-021-01214-y

**Published:** 2021-11-22

**Authors:** Nagendra Awasthi, Clifford Liongue, Alister C. Ward

**Affiliations:** 1 School of Medicine, Deakin University, Pigdons Road, Geelong, VIC 3216 Australia; 2 Institue of Mental and Physical Health and Clinical Translation (IMPACT), Deakin University, Geelong, VIC Australia

**Keywords:** STAT, JAK, Cytokine, Transcription factor, Immunity, Cancer

## Abstract

STAT proteins represent an important family of evolutionarily conserved transcription factors that play key roles in diverse biological processes, notably including blood and immune cell development and function. Classically, STAT proteins have been viewed as inducible activators of transcription that mediate cellular responses to extracellular signals, particularly cytokines. In this ‘canonical’ paradigm, latent STAT proteins become tyrosine phosphorylated following receptor activation, typically via downstream JAK proteins, facilitating their dimerization and translocation into the nucleus where they bind to specific sequences in the regulatory region of target genes to activate transcription. However, growing evidence has challenged this paradigm and identified alternate ‘non-canonical’ functions, such as transcriptional repression and roles outside the nucleus, with both phosphorylated and unphosphorylated STATs involved. This review provides a revised framework for understanding the diverse kaleidoscope of STAT protein functional modalities. It further discusses the implications of this framework for our understanding of STAT proteins in normal blood and immune cell biology and diseases such as cancer, and also provides an evolutionary context to place the origins of these alternative functional modalities.

## Background

The signal transducer and activators of transcription (STAT) family of proteins were initially identified as transcription factors that could facilitate the rapid induction of target genes in response to specific extracellular stimuli, including cytokines, growth factors and other agents, through tyrosine phosphorylation-mediated activation, much of it mediated by Janus kinases (JAKs) [1–5]. These genes impact key cellular processes, including differentiation, proliferation, survival and functional activation [6]. Gene ablation studies of the various STATs have identified essential roles particularly in blood and immune cell development and function (Table 1), but also in mammopoiesis, lactation, postnatal growth and a variety of homeostatic processes.

**Table 1:** Functions of mammalian STAT proteins in immunity

STAT protein	Major functions	References
STAT1	Immunity against viral and bacterial infection	[7, 8]
STAT2	Immunity against viral and bacterial infection	[7, 8]
STAT3	Regulation of innate immunity and inflammation, stem cell maintenance, cell metabolism	[9, 10]
STAT4	Development and function of adaptive and innate immune cells	[11, 12]
STAT5A/B	Development of multiple blood and immune cell lineages	[13]
STAT6	Regulation of innate and humoral immunity	[14, 15]

More recent studies, however, have uncovered alternative STAT functions that lie outside this ‘canonical’ paradigm of transcriptional activation including gene repression [16] and non-nuclear roles [17], as well as functions not requiring tyrosine phosphorylation [18], collectively termed ‘non-canonical’ signaling. This review describes the various STAT protein functionalities and places these in a robust framework that captures the diversity of STAT roles in the normal biology of blood and immune cells and relevant diseases, placing this diversity in an evolutionary context.

## Structure and function of STAT protein family

**Fig. 1:**
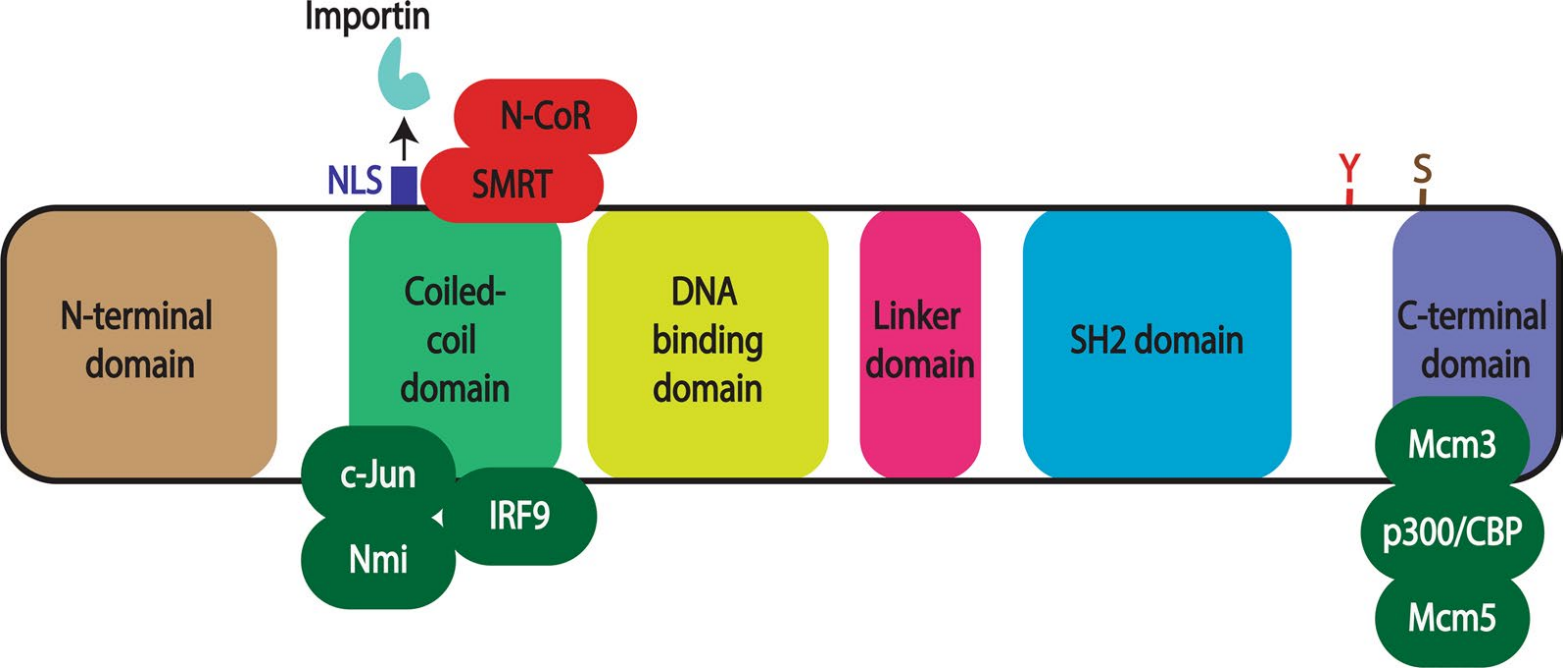
Structure of STAT proteins. Schematic illustration of a representative STAT protein showing its six conserved functional domains: N-terminal, coiled-coil, DNA-binding, linker, Src-homology 2 (SH2) and C-terminal. The positions of a nuclear localization signal (NLS) and tyrosine (Y) and serine (S) residues phosphorylated in response to extracellular stimuli are shown, along with the sites of interaction of various transcriptional co-activators (green) and co-repressors (red)

The STAT protein family consists seven members: STAT1, STAT2, STAT3, STAT4, STAT5A, STAT5B and STAT6 [19, 20]. Each is approximately 750–900 amino acid residues [21] comprising six conserved domains that mediate different aspects of STAT function [22] (Fig. 1).

### N-terminal domain

Consisting of multiple alpha-helices that form a hook-like structure, the N-terminal domain (NTD) mediates interactions between STAT molecules that facilitate dimerization even in the absence of phosphorylation [23, 24].

### Coiled-coil domain

Containing several alpha-helices in a ropelike structure, the coiled-coil domain (CCD) facilitates binding to other transcription factors and co-activators such as p48/Interferon regulatory factor 9 (IRF9) and N-Myc and STAT interactor (Nmi) [25], but also to co-repressors such as Silencing mediator for retinoic acid receptor and thyroid hormone receptor (SMRT)/nuclear receptor co-repressor (N-CoR) [26]. This domain is also implicated in nuclear translocation via a nuclear localization signal (NLS) motif that interacts with importin proteins to facilitate nuclear entry [22, 27].

### DNA-binding domain

The DNA-binding domain (DBD) possess an immunoglobin-like structure that mediates recognition and binding to specific DNA target sequences [22, 28]. Many of these conform to the so-called gamma-activated sequence (GAS), a palindromic TTCN_3-4_GAA motif present within the promoter region of STAT-responsive target genes [6, 29].

### Linker domain

The short linker domain (LD) is crucial for providing structural support during activation and DNA binding, and also serves as a contact point during the formation of transcriptional complexes [22, 30].

### Src homology 2 domain

The Src homology2 (SH2) domain is a highly conserved structural module found in a myriad of signaling proteins that is able to bind to specific phosphotyrosine-containing motifs on other signaling components to mediate protein–protein interactions [31]. In the case of STATs, this includes phosphotyrosines found on activated receptor complexes as well as those found adjacent to the SH2 domain on STAT proteins that facilitate dimerization [1, 20, 22].

### C-terminal domain

A diverse and poorly defined sequence located at the C terminus, this domain is often referred to as the transactivation domain (TAD), since it interacts with numerous transcriptional co-activators such as histone modifying acetyl transferases p300/CREB-binding protein (CBP) [32] and general control of amino acid synthesis protein-5 (GCN5) [33], and the chromatin remodeling factor Brahma [34] to activate gene transcription [35]. A serine residue present within the C-terminal domain undergoes phosphorylation independent of tyrosine phosphorylation and can enhance transcriptional activation [19].

## Canonical STAT function

**Fig. 2:**
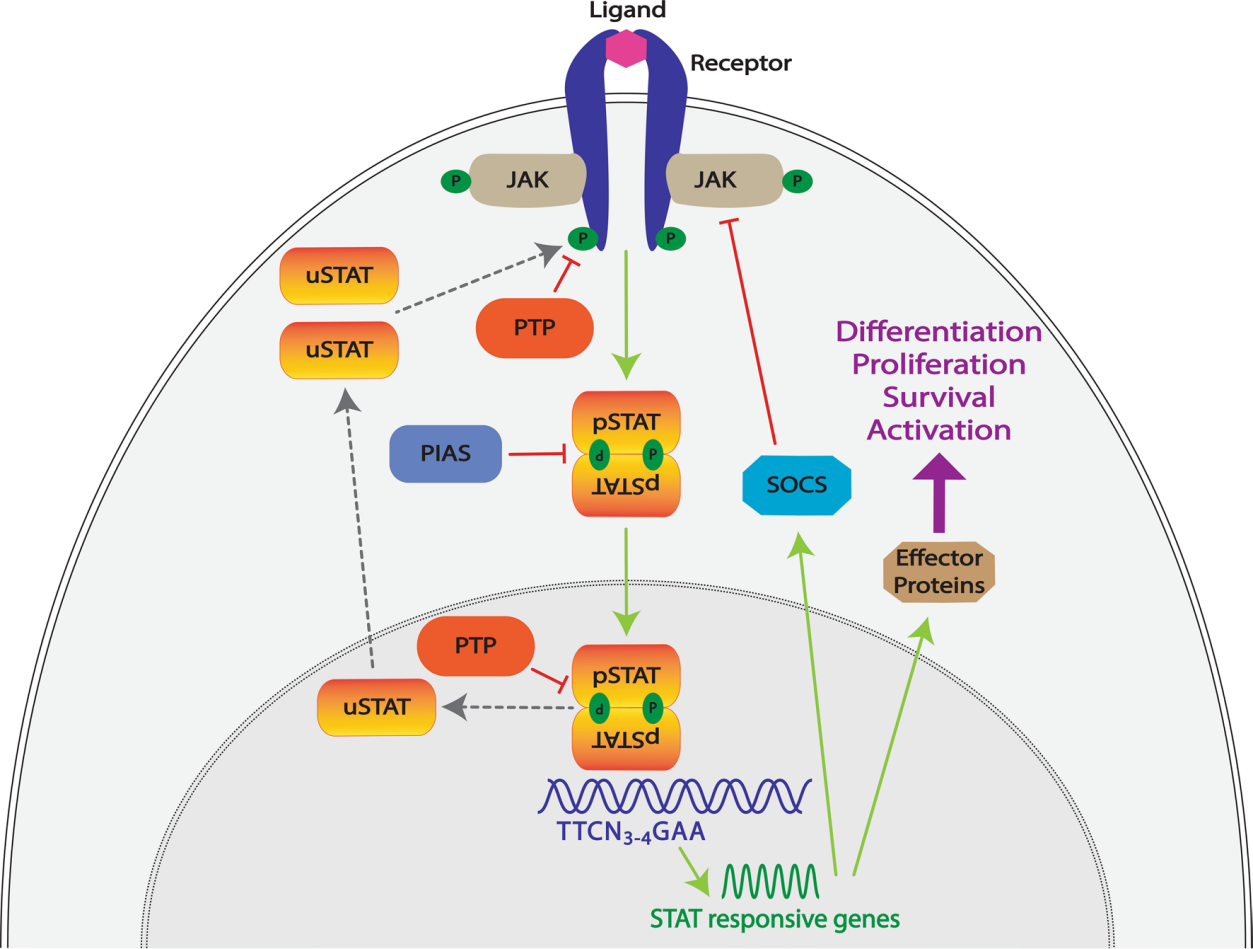
Canonical STAT mode of action. Schematic representation of the archetypal ‘canonical’ STAT functional modality and its control. In this paradigm, STAT proteins (orange/yellow) exist in the cytoplasm as latent, unphosphorylated STAT (uSTAT) molecules. In response to binding of their cognate extracellular ligands (light pink), transmembrane receptors (dark blue) undergo conformational changes that results in the activation of kinases such as the receptor-associated JAKs (light brown), which subsequently mediate phosphorylation (P, green) of tyrosine residues within the intracellular receptor complex, thereby creating docking sites for signaling molecules, including uSTATs. These in turn become tyrosine phosphorylated, with the phosphorylated (pSTAT) molecules able to form dimers that can translocate into the nucleus and bind to specific DNA sequences (blue) to activate the transcription of responsive genes. These encode effector proteins (brown) responsible for cell differentiation, proliferation, survival and activation, as well as SOCS proteins (blue). These mediate a negative feedback loop by blocking STAT activation through interfering with STAT docking, inhibiting JAKs and/or mediating degradation of receptor signaling components. Other negative regulators include PIAS proteins (grey blue) that act via blocking STAT dimerization and nuclear entry and Protein tyrosine phosphatase (PTP) proteins (orange) that can dephosphorylate receptor complex components in the cytoplasm as well as pSTAT molecules in the nucleus to regenerate uSTAT molecules that return to the cytoplasm

In the well-established ‘canonical’ STAT functional mode (Fig. 2), unphosphorylated STATs (uSTATs) reside in an inactive state in the cytoplasm and require tyrosine phosphorylation to become active.

### Receptors

Extracellular regulators, such as cytokines and growth factors, bind to specific transmembrane receptors present on the plasma membrane of responsive cells. As a result, the intrinsic tyrosine kinase domains found in growth factor receptors and associated non-receptor tyrosine kinases, such as Janus kinases (JAKs), in the case of cytokine receptors become activated.

### JAKs

JAKs associate with the membrane proximal region of cytokine receptors via interaction with conserved sequence motifs [36]. Cytokine-induced conformational changes in the receptor complex facilitate JAK activation through autophosphorylation, with activated JAKs able to phosphorylate tyrosine residues within the cytoplasmic domain of the receptor and associated molecules [37]. This creates docking sites for downstream signaling proteins containing SH2 domains, including uSTAT molecules [2, 31, 38].

### STATs

Once docked to the receptor complex the uSTATs in turn become tyrosine phosphorylated, with the resultant phospho-STAT (pSTAT) able to form a dimer with another pSTAT via reciprocal SH2 domain–phosphotyrosine interactions, which can rapidly translocate into the nucleus by direct interaction with importin complexes in the nuclear membrane mediated by GTPases such as Rac1 [7, 39, 40] Within the nucleus the pSTAT dimers bind to specific DNA sequences in the promoter region of target genes typically based on variations of a core palindromic TTCN_3-4_GAA motif [6, 19]. This results in the transcriptional activation of these genes via associated co-activators [35]. The target genes encode proteins that are associated with various cellular activities such as proliferation, differentiation, survival and activation [6].

### Negative regulators

STATs subsequently undergo inactivation by dephosphorylation via nuclear protein tyrosine phosphatases (PTPs) and are exported back to the cytoplasm [18, 41]. Canonical STAT function is also negatively regulated by protein inhibitors of activated STAT (PIAS) protein through direct binding to STAT proteins to suppress nuclear entry and DNA-binding activity, cytoplasmic PTPs that dephosphorylate various receptor components to inhibit STAT activation [42], and by members of the SOCS family of negative feedback regulators, which are induced by STAT signaling and then serve to inhibit further signaling via a number of mechanisms [43, 44].

### Variations

Even within this ‘canonical’ mode, there are variations, such as the formation of a STAT1/STAT2/IRF9 hetero-trimeric complex in response to type I interferons [45], which targets an alternative sequence, the interferon-sensitive response element (ISRE: YAGTTC(A/T)TTTYCC) [46]. There is also evidence of other heterodimeric combinations, such as STAT1/IRF9, STAT1/STAT3, STAT3/STAT5 and STAT5A/STAT5B [47–50]. In addition, multimeric sites comprising more than one STAT dimer binding cooperatively to enhance the impact on transcription have been described [51, 52].

## A new framework to describe alternative STAT functional modalities

**Fig. 3:**
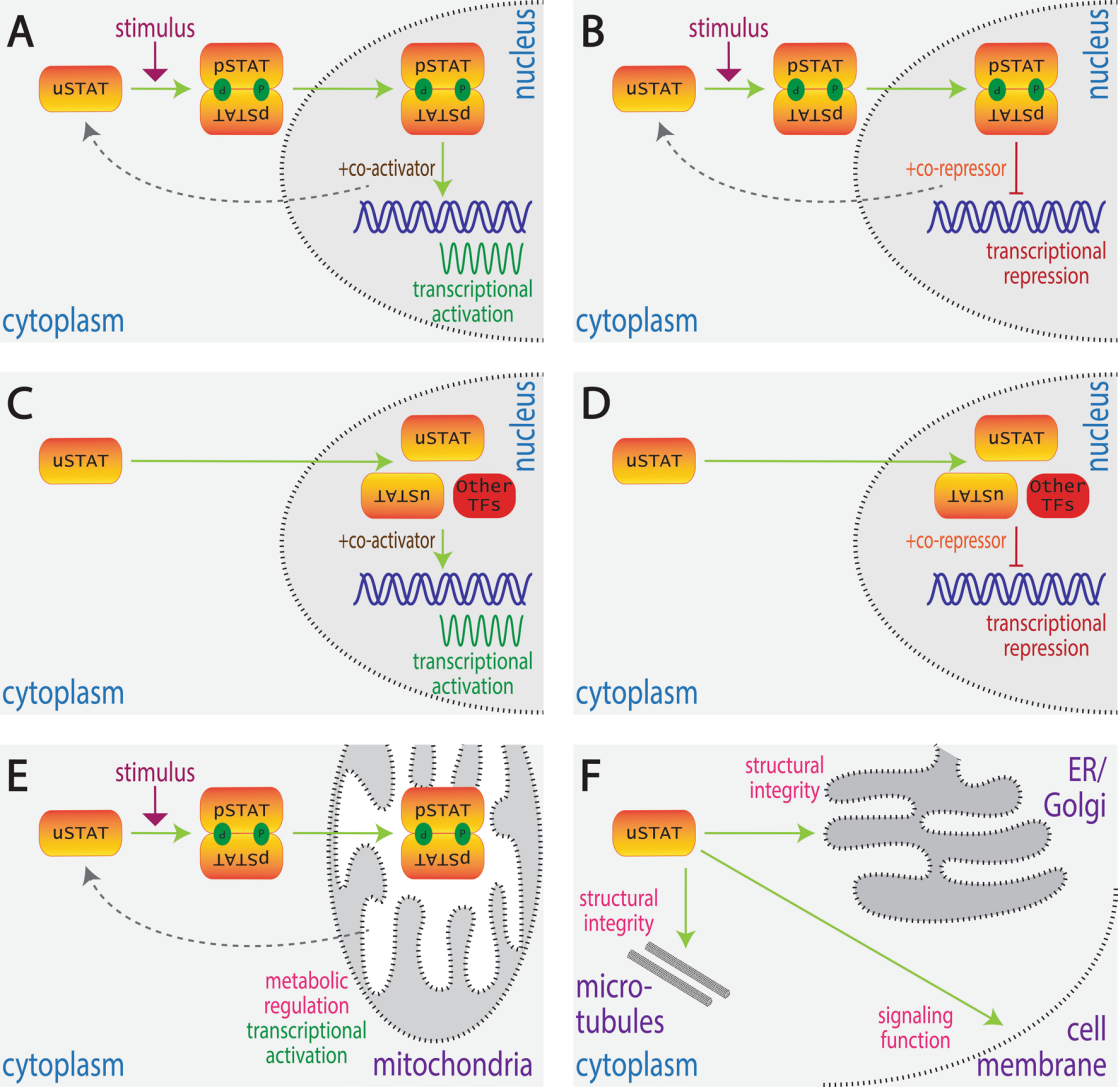
Alternative STAT functional modalities. Schematic depiction of alternate modes by which STATs can impact on cellular functions: **A** inducible transcriptional activation (‘canonical’ signaling), **B** inducible transcriptional repression, **C** basal transcriptional activation, **D** basal transcriptional repression, **E** inducible non-nuclear function, **F** basal non-nuclear function. Shown are unphosphorylated STAT (uSTAT) molecules and their conversion into phosphorylated STAT (pSTAT) molecules and their dimerization where appropriate, as well as their movement between the cytoplasm, nucleus and other cellular compartments, along with the molecular function(s) that they exert in each case

There is growing evidence that STATs can exert their effects via additional modalities distinct from the canonical mode. Collectively these alternative modalities, termed ‘non-canonical’ [16], utilize a variety of different mechanisms, including both gene repression and activation, mediated by both tyrosine phosphorylated and unphosphorylated STAT proteins, and involving roles both inside and outside the nucleus. This review provides an overarching framework to describe these modalities to facilitate a greater coherence to the literature and as a necessary prelude to defining the specific function(s) of each modality (Fig. 3).

### Nuclear functions

The predominant function for STAT proteins is still regarded to be as a transcriptional regulator in the nucleus, but several variations of this have now been described. In addition to transcriptional activation, pSTATs have also been shown to mediate repression of target genes, for example STAT5 during embryonic erythropoiesis [53]. Moreover, it is now well established that uSTATs are capable of entering the nucleus to regulate gene expression through either activation or repression [16]. These can represent cytoplasmic uSTAT dimers that translocate into the nucleus, which for uSTAT3 involves importins [54], but for uSTAT1 is mediated by direct interaction with nucleoporins [55]. Alternatively, they may be uSTATs that result from dephosphorylation of nuclear pSTAT molecules [56]. Such uSTATs can bind similar, overlapping or totally distinct sites to the corresponding pSTAT [57, 58]. For example, uSTAT3 has been shown to preferentially bind with AT-rich DNA sequences and specific DNA structures, leading to the heterochromatin formation resulting in the gene silencing [59].

#### Inducible transcriptional activation

This represents the classical ‘canonical’ mode of signaling utilized by all STATs (Fig. 3A, Table 2), in which uSTAT proteins are dormant in the cytoplasm and then become activated by tyrosine phosphorylation in response to cytokines [1], growth factors [3], G-protein-coupled receptors [60] or other stimuli such as osmotic stress [61]. Tyrosine phosphorylation allows dimerization and subsequent translocation of pSTAT dimers into the nucleus to activate target genes via interaction with co-activators such as CREB-binding protein (CBP), p300 and NCoA-1/SRC-1 [35, 62], as well as chromatin remodelers such as BRG1 [63] until pSTAT molecules are inactivated by dephosphorylation [18, 41].

**Table 2:** Canonical and non-canonical functions of different STAT proteins

Functional modality	STAT protein	Examples	References
Inducible transcriptional activation	STAT1 and STAT2	Type I IFN stimulated pSTAT1/pSTAT2/IRF9 leads to transcription of IFN-stimulated genes (ISGs) that provide protection against viral infection	[64, 65]
	STAT3	IL-6 stimulated pSTAT3 leads to transcription of *IL21* and other genes essential for Th17 cell differentiation	[66]
	STAT4	IL-12 stimulated pSTAT4 leads to transcription of *NFKB1*, *CASP8* and other genes that impact neutrophil function	[12, 67]
	STAT5	EPO stimulated pSTAT5 leads to transcription of genes like *GATA1*, *KLF1* and *BCL2* that are essential for erythropoiesis	[68]
	STAT6	IL-4 stimulated STAT6 leads to transcription of genes like *IL4*, *GATA3*, *IL1R11*, *HIPK2* and *NFIL* that are responsible for Th2 cell differentiation	[69]
Inducible transcriptional repression	STAT5	IL-7 stimulated pSTAT5 leads to repression of *IGK* that suppresses immunoglobulin gene rearrangement	[70]
	STAT6	IL-4 stimulated pSTAT6 leads to repression of genes like *NLRP3* and *IL1B*, facilitating alternative macrophage polarization	[71]
Basal transcriptional activation	STAT1	uSTAT1/IRF1 heterodimer mediates transcription of *LMP2*	[57]
	STAT1 and STAT2	uSTAT1/uSTAT2/IRF9 mediates prolonged transcription of IFN-responsive genes	[18]
	STAT3	uSTAT3/uNFκB mediates transcription of *RANTES*, *IL6*, *IL8*, *MET* and *MRSA*	[72, 73]
Basal transcriptional repression	STAT5	uSTAT5/CTCF complex represses expression of genes involved in megakaryocytic differentiation	[58, 74]
Inducible non-nuclear functions	STAT3	IL-6 stimulated pSTAT3 alters Ca2 + levels and membrane potential in mitochondria to influence effector functions of CD4 + T-cells	[75]
	STAT5	IL-2 stimulated pSTAT5 regulates mitochondrial gene expression	[76]
Basal non-nuclear functions	STAT1	uSTAT1 functions at immunological synapses in NK cells to impact cytotoxicity	[77]
	STAT3	uSTAT3 sequesters FoxO transcription factors within cytoplasm that extends T cell activation	[78]
	STAT5	uSTAT5 maintains structural integrity of endoplasmic reticulum, Golgi body and mitochondria	[79]

Numerous cytokines have been shown to utilize STATs in this manner leading to induction of a raft of genes involved in specific cellular functions along with proliferation, differentiation and survival [6]. For example, IFNγ signaling leads to the phosphorylation, dimerization and nuclear translocation specifically of pSTAT1 to activate various genes such as various *IRF*, *CXCL* and *ISG* genes and micro RNAs essential for immune responses to pathogen and tumors via so-called γ-activated sites (GAS) motifs within their promoters [18, 80, 81]. Alternatively, IL-6 induces the transcription of *IL21* via pSTAT3 in T cell precursors, with the encoded IL-21 in turn inducing *IL17* that is essential for Th17 differentiation also via pSTAT3 activation [66]. Similarly, IL-12 induces pSTAT4 in CD3^+^ T cells that induces genes such as *IFNγ* that impact on cell differentiation and function [82]. In addition, in the erythroid lineage erythropoietin (EPO) to induce genes such as *BCL2l1* and *TRAF5* via pSTAT5 in concert with KLF-1 and GATA1 to regulate erythropoiesis [68].

#### Inducible transcriptional repression

In this alternative modality, dormant cytoplasmic uSTAT proteins are also tyrosine phosphorylated in response to external stimuli and form pSTAT dimers that move to the nucleus and bind to specific DNA sequences, but instead actively repress transcription of certain target genes (Fig. 3B, Table 2). This is facilitated via interaction with co-repressors such as Silencing mediator for retinoic acid receptor and thyroid hormone receptor/nuclear co-repressor 2 (SMRT/Ncor2) that induce histone modification [26]. The pSTAT dimers involved would also be inactivated over time to remove the repressive effect. Numerous examples of this modality have been described. For example, IL-4-induced pSTAT6 mediates the repression of various genes involved in the alternative macrophage polarization, such as *NLRP3* and *IL1B*, thereby inhibiting inflammasome stimulation and pyroptosis [71]. Similarly, IL-7-induced pSTAT5 binds to intronic tetrameric STAT sites in B cells to repress the transcription of the *IGK* gene to suppress immunoglobin gene rearrangement [70], while IL-2-induced pSTAT5 suppresses a Th17-like program during Th9 cell differentiation [83].

#### Basal transcriptional activation

In this functional mode, nuclear uSTAT molecules mediate the transcriptional activation of certain target genes, often through the formation of novel complexes with other transcription factors [35]. In response to external signals, the levels of pSTAT become acutely increased, which serves to indirectly deplete levels of uSTAT that results in an acute deactivation of uSTAT-activated genes (Fig. 3C, Table 2). For example, uSTAT1 can form a heterodimeric complex with interferon regulatory factor-1 (IRF1) that can bind GAS motifs to induce genes such as *LMP2* that encodes a subunit of the 20S proteosome [57]. In addition, uSTAT1 has been shown to activate expression of pro-apoptotic genes, such as caspases *CPP22*, *ICE* and *ICH1* necessary for TNFα-mediated apoptosis [84]. In a similar vein, uSTAT3 mediates induction of ion channels and neurotransmitter receptors in the brain [85] and can also augment the expression of select STAT1- and STAT2-responsive genes by increasing promoter accessibility [86]. However, in several cases ‘canonical’ signaling can also increase the transcription of the respective *STAT* gene, thereby elevating uSTAT levels that leads to sustained basal transcriptional activation over a longer time frame. For example, via a complex of uSTAT1, IRF9 and uSTAT2 resulting in prolonged expression of a subset of IFN-induced genes [18], or in the case of uSTAT3 to induce a second wave of alternate genes [72].

#### Basal transcriptional repression

In this modality, uSTAT molecules can repress transcription of specific target genes via interaction with co-repressors and/or chromatin modifiers, including heterochromatin protein (HP)1α that mediates heterochromatin formation [87]. In response to external signals, acute levels of these uSTAT molecules tend to decrease leading to derepression of these genes (Fig. 3D, Table 2). For example, uSTAT5 can act as a direct repressor of genes via the transcription repressor CTCF to restrain megakaryocytic differentiation. However, thrombopoietin (TPO)-stimulated STAT5 phosphorylation results in decreased levels of uSTAT5 thereby abrogating the repression of these genes [58, 74].

### Non-nuclear roles

STATs have also been implicated in various non-nuclear roles, including in mitochondria, endoplasmic reticulum (ER), Golgi apparatus and cytoplasm in both unphosphorylated and phosphorylated states, the majority not involving gene regulation [17, 88].

#### Inducible non-nuclear functions

In this mode, dormant uSTAT is activated in response to external stimuli and moves into non-nuclear organelles to exert various biological roles (Fig. 3E, Table 2). For example, IL-6-mediated mitochondrial translocation of STAT3 has been shown to mediate cytokine expression in CD4 + T cells [75], while mitochondrial STAT3 induced by IL-21 contributed to the generation of CD8 + memory T cells and antibody production in B cells [89]. IL-2 has also been shown to induce translocation of pSTAT5 into mitochondria, where it was able to associate with the D-loop regulatory region of mtDNA, suggesting it may also participate in the regulation of mitochondrial DNA transcription [76].

#### Basal non-nuclear functions

In this remaining modality, uSTAT molecules exert a function outside of the nucleus (Fig. 3F, Table 2), with the impact of external signals currently unclear. For example, uSTAT1 functions at immunological synapses in NK cells to contribute to cytotoxicity [77]. Meanwhile uSTAT3 sequesters the FoxO transcription factors in the cytoplasm to prolong T cell activation, which is released by pro-inflammatory cytokines that convert it to pSTAT3 [78]. uSTAT3 has also been shown to be involved in the regulation of microtubule structural integrity in murine embryonic fibroblast cells through its antagonistic association with the cytoplasmic microtubule destabilizing protein stathmin [90]. Alternatively, uSTAT5A and uSTAT5B have been identified in the ER and Golgi apparatus in human pulmonary arterial endothelial and smooth muscle cells, where they contribute to the anterograde vesicular secretory pathway. Knockdown of STAT5A/B resulted in dilation and/or fragmentation of the ER, Golgi and mitochondria, along with effects on other organelles [79].

## Implications of this framework

This framework provides a powerful lens with which to view the various studies that address STAT functions, allowing a fresh and more complete perspective of STATs in both normal biology and disease.

### Reassessment of the current literature

The majority of studies to date examining STAT function have utilized ‘global’ knockout/knockdown strategies, which provide excellent information about the overall function of the individual STAT proteins [91]. However, the key limitation of such approaches is that they do not provide detail about which specific STAT signaling modality is responsible for particular phenotypic changes. More problematic, however, has been the inherent assumption in many cases that the alterations observed relate exclusively to the loss of the canonical/inducible transcriptional activation mode. Therefore, the potential role of the various non-canonical modalities should also be directly considered. This will require alternative methodological approaches using more specific gene modifications that selectively target particular modalities, as already performed in a number of breakout studies [73, 86, 92].

### More comprehensive insight into STAT biology

This framework also helps understand the full gamut of responses to cytokines and growth factors, rather than just the acute effects. Perhaps the best documented examples relate to interferon signaling where our understanding is unparalleled [93]. Thus, it is known that in the absence of IFNs, uSTAT-containing complexes facilitate low basal expression of a core set of genes [94]. IFN signaling then induces high level, but transient canonical pSTAT-mediated expression of various target genes that serve to inhibit growth and promote apoptosis and immune surveillance genes [94]. However, the targets also include *STAT1*, *STAT2* and *IRF9* [95]. A few hours after IFN stimulation, pSTAT levels decline, but those of uSTAT become increased as a combined consequence of pSTAT de-phosphorylation and increased *STAT* gene expression, allowing for a sustained impact on the transcription of a different set of immune genes that elicit long-lived antiviral and immune responses [18]. Similarly, it has been shown that IL-6 can increase *STAT3* expression in fibroblasts (through canonical signaling via pSTAT3), which ultimately results in increased levels of uSTAT3 that drives the expression of late phase genes such as *CDC2*, *CCNB1* and *E2F11* [72, 73]. Finally, with respect to megakaryocyte differentiation, it has been demonstrated that in the absence of TPO uSTAT5 blocks differentiation by repressing the genes responsible for this process, including *MPL* and *FTR*. However, TPO-stimulated pSTAT5 phosphorylation results in decreased levels of uSTAT5 and so the repression of these genes is removed in concert with the induction of genes by pSTAT5 promoting proliferation and survival [58, 74].

### More nuanced understanding of relevant human diseases and their potential treatment

STAT proteins have been widely implicated in human diseases, particularly immune deficiencies, inflammatory diseases, cancers and other proliferative disorders [96–98]. However, the current understanding of disease etiology has primarily focused on impacts to canonical signaling. This framework allows a recalibration of STAT-mediated diseases such that the full gamut of non-canonical functionalities can also be considered (Table 3).

**Table 3:** Role of different STATs in cancer and immune diseases

Functional modality	STAT protein	Disruption*	Clinical manifestations	References
Inducible transcriptional activation	STAT1	Germline LoF mutation	Susceptibility to intracellular pathogens and herpetic infection, due to defective IFN responses	[99]
		Germline GoF mutation	Mucocutaneous candidiasis, recurrent respiratory infection, cancer, autoimmune cytopenias, due enhanced responses to IFNs and other cytokines	[100]
	STAT2	Germline LoF mutation	Susceptibility to viral disease, due to defective IFN responses	[101]
		Germline GoF mutation	Various autoinflammatory disorders, due to enhanced IFN responses	[100]
	STAT3	Germline LoF mutation	Hyper-IgE syndrome, with cutaneous and respiratory infections and skeletal abnormalities, due to defective signaling by multiple IL-6-related cytokines	[99]
		Germline GoF mutation	Multiorgan autoimmunity, short stature, lymphoproliferation, due to enhanced signaling by IL-6 and related cytokines	[100]
		Acquired activating mutation	Large granular lymphocytic (LGL) leukemia and diffuse large B cell leukemia (DLBCL), due to augmented proliferation and survival	[102]
		Hyperactivation due to upstream components	Various cancers, such as head and neck squamous cell carcinoma, due to induction of genes such as *CCND1* and *TERT* that permit sustained proliferation	[103–105]
	STAT4	Germline GoF mutation	Autoimmune disorders, due enhanced signaling by IL-12 and other cytokines	[106]
	STAT5A	Acquired activating mutation	T cell leukemias, due to increased proliferation and survival	[107]
	STAT5B	Germline LoF mutation	Growth hormone insensitivity, immunodeficiency, eczema	[99]
		Acquired activating mutation	Multiple T cell leukemias, due to increased proliferation and survival	[102]
	STAT5	Hyperactivation due to upstream components	Myeloproliferative neoplasms such as acute myeloid leukemia (AML), chronic myeloid leukemia (CML), due to increased proliferation and survival	[108]
Inducible transcriptional repression	STAT1	Loss of expression	Various cancers (melanoma, oesophageal squamous cell carcinoma, lung cancer, breast cancer), due to disruption of tumor suppressor activity of STAT1	[109, 110]
	STAT3	Hyperactivation due to upstream components	Chronic lymphocytic leukemia, due to increased repression of *TP53* tumor suppressor gene	[72, 111]
	STAT5	Loss of STAT5-binding sites	Various B cell malignancies caused by constitutive expression of BCL6A that is normally repressed by STAT5	[112, 113]
Basal transcriptional activation	STAT3	Overexpression due to upstream components	Various cancers due to increased expression of oncogenes such as *CCNB1* and *E2F11* via excessive uSTAT3	[73]
	STAT6	Overexpression due to upstream components	Hepatocellular carcinoma due to increased cyclooxygenase-2 expression via excessive uSTAT6	[114, 115]
Inducible non-nuclear roles	STAT5	Hyperactivation due to upstream components	Interacts with scaffold adaptor to mediate cell survival and metabolism of cancer cells	[76, 116]

*GoF, gain of function; LoF, loss of function

This is not to say that canonical STAT signaling resulting in inducible gene activation is not important in disease. Indeed in numerous cancers and proliferative disorders, STAT proteins have been observed to be constitutively phosphorylated as a result of mutation of upstream activators, or through excessive signaling associated with inflammation, infection or other pathologies [117–120]. This is typically associated with an increase in the expression of genes usually induced through the canonical pathway. For example, in various human cancers STAT3 induces genes that permit sustained proliferation, such as those encoding cyclin D1 (*CCND1*) [103] and telomerase (*TERT*) [104]. However, there is also evidence for inducible transcriptional repression. For example, IL-2-mediated pSTAT3 represses p53 to enhance survival of chronic lymphocytic leukemia cells [111]. Similarly, in mammary epithelial cells prolactin-mediated pSTAT5A results in repression of the *BCL6A* gene through binding to tetrameric STAT sites within the 5’ untranslated exon of this gene to decrease breast cancer tumorigenesis [121].

Unphosphorylated STATs also play a role in disease. This can involve basal transcriptional activation. For example, in non-small cell lung cancer uSTAT6 upregulates the cyclooxygenase 2 gene (*PTGS2*) through a consensus STAT6 site to provide protection against apoptosis [114]. One of the targets of canonical STAT3 signaling is the *STAT3* gene itself, which serves to increase the levels of uSTAT3 as well over the longer term, resulting in chronic pSTAT3-mediated transcriptional effects in concert with additional uSTAT3-mediated regulation. In lung, head and neck cancers, this includes genes involved in cell cycle progression, such as *CCNB1*, *E2F11* and *CDC7* [73]. Importantly, genes not typically regulated by pSTAT3 can be affected, such as those encoding cytokines (*IL6*, *IL8*, *RANTES*) and oncoproteins (*MRAS*, *MET*) in hTERT-HME1 cells. Expression of these genes was shown to be mediated by a novel transcription complex formed when uSTAT3 binds unphosphorylated NFκβ (uNFκβ) [72]. Similarly, in colon cancer, Jun activation domain-binding protein 1 (Jab1) regulates uSTAT3 DNA binding and expression of *VEGF*, *MDR1* and *NANOG* [122]. However, the basal repression modality of uSTATs is also important. For example, uSTAT3 and uSTAT5A have been shown to interact with HP-1α, promoting the formation of heterochromatin which contributes to gene silencing that suppresses growth of colon and lung cancer cells, respectively [123, 124].

In addition, non-nuclear roles have been implicated in disease, including cancer, which can be inducible or basal. For example, pSTAT5 induced by BCR-ABL, JAK2^V617F^ or KIT^D816V^ in leukemic cells has been shown to interact with the scaffold adaptor Gab2 in the cytoplasm to mediate activation of the phosphatidylinositol-3 kinases (PI3K)/AKT pathway to facilitate cell survival [116]. Alternatively, mitochondrial STAT5 and has been found to interact with the E2 subunit of the pyruvate dehydrogenase complex to regulate metabolism in leukemic T cells [76]. In leukemic pre-B cells uSTAT5 protects against oxidative damage independent of transcriptional changes [51]. The pathogenesis of pulmonary arterial hypertension has also been proposed to be mediated by non-nuclear functions of STAT3 [125] and STAT5 [17], which appear to impact on the stability of the ER and Golgi body [79].

These examples clearly indicate that relevant diseases involve both canonical and non-canonical modalities. This knowledge provides critical insights for the design and application of therapeutic agents targeting STAT proteins. This is particularly important since most of the developed pharmacological inhibitors target either upstream canonical signaling components such as the kinase activity of JAKs or the SH2 domains of STATs [121] (Table 4). However, these drugs may not be effective on the non-canonical pathways. Moreover, there is a possibility for negative consequences, with inhibition of pSTAT potentially resulting in accumulation of uSTAT that may exert unintended and potentially deleterious effects.

**Table 4:** Examples of inhibitors of STAT pathway components in clinical trials for cancer and immune-related disorders

Agent	Target(s)	Disease(s)	Phase	Status*	ClinicalTrials.gov identifier(s)
CPL409116	JAKs/ROCK	Rheumatoid arthritis, psoriasis	1	R	NCT04670757
Abrocitinib (PF-04965842)	JAK1	Atopic dermatitis	3	C, C	NCT04345367, NCT03796676
		Food allergy	1	N	NCT05069831
GSK2586184	JAK1	Psoriasis	2	C	NCT01782664
		Systemic lupus erythematosus	1	R	NCT01953835
Itacitinib (INCB039110)	JAK1	Rheumatoid arthritis	2	C	NCT01626573
		Plaque psoriasis	2	C	NCT01634087
		Myeloproliferative neoplasms	2	C, C	NCT03144687, NCT01633372
		Non-small cell lung cancer	2	A	NCT03425006
		Graft versus host disease	2	R	NCT04200365
		Hemophagocytosis lymphohistiocytosis	2	N	NCT05063110
		B-cell lymphoma	1/2	C, A	NCT02018861, NCT02760485
		Leukemia (acute myeloid, acute lymphocytic, myelodysplastic syndrome)	1	R	NCT03755414
		T-cell leukemia	1	R	NCT03989466
		Hepatocellular carcinoma	1	R	NCT04358185
		Sarcoma	1	R	NCT03670069
SHR0302	JAK1	Rheumatoid arthritis	3	R	NCT04333771
		Ankylosing spondylitis	2/3	R	NCT04481139
		Atopic dermatitis	2/3	R	NCT04717310
		Ulcerative colitis	2	C	NCT03675477
		Crohn's disease	2	C	NCT03677648
Upadacitinib	JAK1	Inflammatory bowel disease (Crohn's disease)	3	A	NCT03345823
		Atopic dermatitis	3	A	NCT04195698
		Axial spondyloarthritis	3	A	NCT04169373
Baricitinib (INCB28050, LY3009104)	JAK1/2	Atopic dermatitis	3	C	NCT03435081
		Juvenile idiopathic arthritis	3	A	NCT03773978
		Sjogren's syndrome	1/2	C	NCT04916756
		Graft versus host disease	1	R	NCT04131738
Momelotinib (CYT387)	JAK1/2	Myelofibrosis (primary, post-polycythemia vera, post-essential thrombocythemia)	3, 2	C, C	NCT02101268, NCT01969838
Jaktinib	JAK1/2	Myelofibrosis (primary, post-polycythemia vera, post-essential thrombocythemia)	2	R	NCT04217993
		Acute graft versus host disease	2	N	NCT04971551
		Atopic dermatitis	1/2	R	NCT04435392
Ruxolitinib (INCB018424)	JAK1/2	Alopecia areata	4	C	NCT03800979
		Myelofibrosis	4	C	NCT01558739
		Acute promyelocytic leukemia	4	R	NCT04446806
		Polycythemia vera	3	C, C	NCT02038036, NCT02292446
		Atopic dermatitis	3	C, R	NCT03745651, NCT04921969
		Graft versus host disease	3	A	NCT03112603
		Vitiligo	3	A	NCT04057573
TLL018	JAK1/TYK2	N/A	1	C	NCT04243083
Tofacitinib (CP-690550)	JAK1/JAK3	Alopecia areata	4	C	NCT03800979
		Ulcerative colitis	4	A	NCT03281304
		Rheumatoid arthritis	3	C	NCT00661661
		Juvenile idiopathic arthritis	3	C	NCT02592434
		Inflammatory eye disease	2	A	NCT03580343
VR588 (KN002)	JAKs	Severe asthma	1	C	NCT02740049
Brepocitinib (PF-06700841)	JAK1/TYK2	Psoriatic arthritis	2	C	NCT03963401
		Systemic lupus erythematosus	2	R	NCT03845517
		Cicatricial alopecia	2	R	NCT05076006
AZD1480	JAK2	Myelofibrosis (primary, post-polycythemia vera, essential thrombocythemia)	1	C	NCT00910728
Gandotinib (LY2784544)	JAK2	Myeloproliferative neoplasms (essential thrombocythemia, polycythemia vera)	1, 2	C, A	NCT01520220, NCT01594723
Fedratinib (SAR302503)	JAK2	Myelofibrosis	2	C	NCT01523171, NCT01420770
		Polycythemia vera, essential thrombocythemia	2	C	NCT03755518, NCT03952039
Ilginatinib (NS-018)	JAK2	Myelofibrosis (primary, post-polycythemia vera)	1/2, 2	C, N	NCT01423851, NCT04854096
Pacritinib (SB1518)	JAK2	Lymphoid malignancy (Hodgkin’s, mantle cell, indolent)	2	C	NCT01263899
		Prostate cancer	2	R	NCT04635059
		Myelofibrosis (essential thrombocythemia, polycythemia vera)	1,2	C	NCT00745550
		Acute myeloid leukemia	1	C	NCT02323607
TQ05105	JAK2	Myelofibrosis	2	N	NCT05020652
		Chronic graft versus host disease	1/2	R	NCT04944043
		Hemophagocytic lymphohistiocytosis	1	R	NCT04326348
		Myeloproliferative neoplasms	1	R	NCT04339400
Decernotinib (VX-509)	JAK3	Rheumatoid arthritis	2, 2/3	C, C	NCT01052194, NCT01830985
Ritlecitinib (PF-06651600)	JAK3	Alopecia areata	2	C	NCT02974868
		Rheumatoid arthritis	2	C	NCT02969044
PF-06826647	TYK2	Psoriasis	2	C	NCT03895372
Danvatirsen (AZD9150)	STAT3	Carcinoma (non-small cell lung, pancreatic)	2	A	NCT01839604, NCT02983578, NCT03421353
		Hepatocellular carcinoma	1	C	NCT01839604
		Lymphoma (non-Hodgkin’s, DLBCL)	1	C	NCT03527147
TTI-101 (C188-9)	STAT3	Cancers (breast, head and neck, non-small cell lung, hepatocellular, colorectal, advanced cancer, squamous cell carcinoma, gastric adenocarcinoma and melanoma)	1	R	NCT03195699
CpG-STAT3 siRNA CAS3/SS3	STAT3	Lymphoma (B-cell—various)	1	R	NCT04995536
WP1066	STAT3	Glioblastoma, melanoma, neoplasm (brain)	1	A, R	NCT01904123, NCT04334863
Napabucasin (BBI608)	STAT3	Gastric and gastroesophageal cancer	3	C	NCT02178956
		Colorectal carcinoma	3	C	NCT01830621
		Pancreatic adenocarcinoma	3	C	NCT02993731
		Glioblastoma	1/2	C	NCT02315534
OPB-51602	STAT3	Advanced cancer	1	C	NCT01423903
		Hematological malignancy (multiple myeloma, non-Hodgkin’s lymphoma, acute myeloid leukemia, acute lymphoblastic leukemia, chronic myelogenous leukemia)	1	C	NCT01344876

*A, active; C, completed; N, not yet recruiting; R, recruiting

## Additional STAT modifications

The framework presented has focused solely on tyrosine phosphorylation and in the context of full-length STAT proteins. However, a range of other modifications of STAT proteins are possible. These are best viewed as mechanisms to further fine tune the STAT response, generally serving to modulate a particular functional modality, for example, phosphorylation of a critical serine residue in the C-terminal domain of STAT1, STAT3, STAT5A and STAT5B, such as STAT5 by IL-2 [126]. This has been shown to positively impact on canonical STAT-mediated gene transcription, such as that induced by hypothalamic STAT3 [127] and mammary STAT5 [128]. For STAT1, this has been shown to be through increased association with histone acetylase complexes [129]. Serine phosphorylation plays a particularly important role in oncogenic STAT signaling [6, 130]. For example, serine phosphorylation of STAT5 was required for BCR-ABL-induced leukemogenesis [123], and phosphorylation of S727 on STAT3 augmented the induction of genes involve in the cellular growth and survival that contributed to progression of chronic lymphocytic leukemia [131]. However, serine phosphorylation can also mediate translocation of STAT3 molecules into mitochondria, during RAS-dependent oncogenic transformation, where it served to negatively regulate the activity of electron transport chain components complex I and complex II by a mechanism that did not require DNA binding or tyrosine phosphorylation [132]. In mammary tumors, pSTAT3 has also been shown to control ER Ca2^+^ flux via interacting inositol 1,4,5-triphosphate receptor (IP3R3) results proteasomal degradation of IP3R3 and inhibits oxidative/ER stress and apoptosis [133].

In addition, acetylation of lysine residues in STAT3 (K685) and STAT5 (K694) by histone acetylases has been described, such in response to cytokines such as IL-6 and prolactin, respectively [134]. Acetylation differentially regulates STATs, impacting on transcriptional activation and protein stability [135]. Acetylation at K685 in uSTAT3 is important in the formation of a stable dimer and its accumulation in the nucleus to regulate target genes [136], with STAT3 shown to be constitutively acetylated at this site in CLL [137]. Contrastingly, SUMOylation of alternative lysine resides, such as K696 in the STAT5A, can antagonize the effect of acetylation and negatively impact canonical STAT signaling through decreased tyrosine phosphorylation [18, 138, 139]. Other chemical modifications have also been reported, including methylation, oxidation and glutathionylation [140–142], but their role in normal STAT biology remains speculative. Finally, truncated versions of several STATs have been described, from either alternative splicing or proteolysis. In general, these are phosphorylated, dimerize, translocate into the nucleus and bind to the putative DNA-binding site in response to cytokines and growth factors. However, instead of transcriptional activation, they often exert a dominant-negative effect through blocking access of transactivation competent STATs and have been also implicated in disease [143–145].

## Evolutionary origins of multiple STAT functional modalities

A key question regarding the multiple STAT functionalities is whether they represent specific ad hoc innovations or instead have deeper evolutionary origins. This is particularly relevant since STATs have a long evolutionary history, predating cytokine receptors and JAKs [146]. Moreover, investigations of STATs in extant primitive species have identified both canonical and non-canonical signaling modalities [147].

Evidence of ‘canonical’ inducible transcriptional activation is evident throughout metazoans, including invertebrates such as *Drosophila melanogaster*. This organism possesses a single STAT known as Stat92E (or Marelle), with a similar structure to mammalian STAT proteins [148, 149]. This is activated downstream of the single *Drosophila* cytokine receptor (Dome) and JAK (Hopscotch) [90], with pStat92E molecules inducing genes associated with immunity and development [150].

However, STAT proteins evolved prior to cytokine receptors and JAKs [146]. For example, the nematode *C. elegans* possesses two STAT-like proteins, STA-1 and STA-2, but no upstream cytokine receptor signaling components [116]. Indeed, STA-2 lacks the tyrosine phosphorylation motif as well as the coiled-coil domain but can act as an inducible transcriptional activator [116, 151]. This is achieved by an alternative mechanism in which epidermal injuries mediates its release from hemidesmosomes allowing it to move to the nucleus to induce the transcription of genes associated with innate immunity [152]. More primitive eukaryotes, such as the slime mold *Dictyostelium discoideum*, also possess STAT-like proteins—again in the absence of upstream cytokine receptor signaling components [147]. *D. discoideum* has four STAT proteins, STATa, STATb, STATc and STATd, composed of coiled-coil, DNA-binding and SH2 domains, but without transactivation domain and N-terminal sequences. STATa is tyrosine phosphorylated in response to extracellular cyclic AMP (cAMP) through the cyclic AMP receptor (cAR1) and translocate into the nucleus to activate genes such as *cudA*, thereby acting as an inducible transcriptional activator [153]. STATc can also become tyrosine phosphorylated and activate expression of genes such as *gapA* and *rtoA* in response to hyperosmotic and other stressors [154]. Finally, plants have also been shown to possess STAT-related GRAS proteins, with highly similar SH2 and DNA-binding domains to other STATs [155], which can act as transcriptional activators to regulate plant development [156, 157].

There is considerable evidence of non-canonical signaling in these organisms as well. For example, *Drosophila* Stat92E has been shown to participate in basal transcriptional repression, with uStat92E able to enter the nucleus and target alternate DNA sites, particularly those involving metabolic and stress-related pathways [150]. This is achieved via an evolutionarily conserved association with HP1 that induces histone methylation and heterochromatin stabilization resulting in gene repression that can enhance genome stability [79] and suppress tumor growth [158]. Dome signaling is able to mediate derepression of these target genes [125]. *C. elegans* STA-1, which shares a similar structure to mammalian STATs but lacks a TAD, also acts as a basal repressor, inhibiting transcription of antiviral genes in the absence of the viral infection [159]. *D. discoideum* STATa similarly functions as an transcriptional repressor of alternative genes such as *ecmB* [153], and serine phosphorylation is also important in the regulation of this STAT, being able to increase its nuclear export [160]. *D. discoideum* STATc can also repress other genes such as *ecmA*, by preventing activator binding [161]. Finally, plant GRAS proteins can function as transcriptional repressors [156, 157].

Collectively these studies imply that both canonical and non-canonical STATs signaling modalities were present early in evolution and have simply been maintained (and further diversified) along the evolutionary path to higher vertebrates, including mammals. Therefore, the framework provided is applicable across all STAT proteins. It should be noted, however, that the classical ‘canonical’ signaling paradigm can best be viewed as a relatively recent innovation within metazoans, coincident with the coalescence of a functional cytokine receptor signaling module in the common precursor of vertebrates and invertebrates [162].

## Conclusion

Our knowledge of STAT proteins has progressed from a simple view of them mediating acute cytokine-mediated transcriptional responses to one where they are involved in a veritable kaleidoscope of functions, many of which impact on immunity and cancer. Notably, many of these modalities are evident in STAT proteins of divergent organisms, suggesting they have early evolutionary origins. However, there remains a gap in knowledge regarding the full breadth of roles mediated by the non-canonical modalities in physiology and disease. This review provides a framework to view these functional modalities that should contribute to filling this gap and provide new insights that will better inform precise therapeutic interventions that target the appropriate modality in disease states.

## Data Availability

Not applicable.

## Abbreviations

CBP: CREB-binding protein
CCD: Coiled-coil domain
DBD: DNA-binding domain
EPO: Erythropoietin
ER: Endoplasmic reticulum
GAS: Gamma-activated sequence
GCN: General control of amino acid synthesis protein
HP: Heterochromatin protein
IRF: Interferon regulatory factor
JAKs: Janus kinase
LD: Linker domain
N-CoR: Nuclear receptor co-repressor
NLS: Nuclear localization signal
Nmi: N-Myc and STAT interactor
NTD: N-terminal domain (NTD)
PAK: P21-activated kinase
PDC: Pyruvate dehydrogenase complex
PIAS: Protein inhibitor of activated STAT
pSTAT: Phosphorylated STAT
PTP: Protein tyrosine phosphatases
SH2: Src homology-2
SMRT: Silencing mediator for retinoic acid receptor and thyroid hormone receptor
SOCS: Suppressor of cytokine signaling
STAT: Signal transducer and activators of transcription
TAD: Transactivation domain
TPO: Thrombopoietin
uSTAT: Unphosphorylated STAT
